# HBP21 Alleviates Sepsis-Induced Acute Kidney Injury by Targeting PI3K/AKT-Mediated M1 Macrophage Polarization

**DOI:** 10.1155/mi/9021628

**Published:** 2025-07-27

**Authors:** Na An, Mingzhi Xu, Ruman Chen, Cuijuan Wang, Yafei Bai

**Affiliations:** Blood Purification Center of Hainan General Hospital, Hainan Affiliated Hospital of Hainan Medical University, Haikou, Hainan 570311, China

**Keywords:** acute kidney injury, HBP21, inflammation, macrophage polarization, PI3K/AKT signaling, sepsis

## Abstract

**Background:** Sepsis-induced acute kidney injury (S-AKI), a life-threatening complication of systemic infection, is driven by macrophage-mediated inflammatory dysregulation. This study explores the role of heat shock binding protein 21 (HBP21) in attenuating renal injury through PI3K/AKT pathway modulation, employing cellular and animal models to dissect its therapeutic mechanisms and clinical relevance.

**Methods:** In vitro, RAW264.7 cells underwent LPS-induced M1 polarization, and HBP21 expression was manipulated to evaluate its role in macrophage phenotype and PI3K/AKT signaling activation. M1/M2 macrophage polarization was quantified by flow cytometry, while coculture with NRK-52E cells evaluated tubular epithelial cell viability (CCK-8) and apoptosis (flow cytometry). An S-AKI rat model was induced via cecal ligation and puncture (CLP). Renal function (serum creatinine [Scr]/blood urea nitrogen [BUN]), tissue damage (hematoxylin and eosin [H&E]/terminal dUTP nick-end labeling [TUNEL]), and inflammation (Western blot/IHC) were systematically analyzed.

**Results:** HBP21 overexpression promoted M2 macrophage polarization and activated PI3K/AKT signaling in LPS-stimulated macrophages. Knockdown of HBP21 obtained the opposite data. Inhibition with LY294002 or activation with 740 Y-P reversed these effects, confirming pathway involvement. Cocultured NRK-52E cells exposed to conditioned medium from HBP21-overexpressing macrophages showed a 62.32% increase in viability and a 56.11% reduction in apoptosis under LPS challenge. HBP21 overexpression in vivo lowered Scr (38.5%) and BUN (47.4%), alleviated tubular damage, and shifted renal macrophages toward an M2 anti-inflammatory phenotype with concurrent TNF-α/IL-6 downregulation.

**Conclusion:** These findings suggest that HBP21 mitigates S-AKI pathogenesis via PI3K/AKT-mediated M2 macrophage polarization, underscoring its translational potential in renal injury therapy.

## 1. Introduction

Sepsis constitutes a severe and unequivocal health threat, arising from an intricate systemic host reaction to infection. It is marked by prolonged immunosuppression in the host, heightened inflammation, and multiorgan dysfunction [1]. Regarding the organs affected by sepsis, the kidney is extremely vulnerable, with acute kidney injury (AKI) incidence exceeding 60% [2]. Currently, the diagnosis of sepsis-induced AKI (S-AKI) predominantly relies on traditional markers like serum creatinine (Scr) and urine output. However, due to their limited sensitivity and accuracy, it is challenging to identify the early stage of S-AKI [3]. Despite standard therapies (antibiotics and fluid resuscitation), S-AKI frequently progresses to systemic organ injury due to insufficient therapeutic efficacy [4]. Consequently, further investigations into the fundamental mechanisms and pathogenesis of S-AKI hold significant importance for early detection and therapeutic intervention in S-AKI.

Current research highlights that excessive inflammatory cascade reactions, a major contributor to kidney damage, significantly influence the progression of S-AKI [5]. Gram-negative bacterial LPS is a standard agent for establishing S-AKI in preclinical studies [6]. In S-AKI, macrophages bridge innate/adaptive immune functions and drive inflammatory cascades [7]. Macrophages adopt M1 (proinflammatory) or M2 (anti-inflammatory) functional states depending on microenvironmental cues [8]. M1 macrophages amplify injury via proinflammatory cytokines and iNOS/ROS-mediated oxidative stress, perpetuating tissue destruction [9]. Conversely, M2 polarization counterbalance inflammation and supports tissue healing through arginase-mediated ECM deposition [10]. Given macrophages' central role in S-AKI pathogenesis, understanding mechanisms driving M1-to-M2 polarization may yield therapeutic targets to mitigate immune-driven injury and accelerate repair.

Heat shock proteins (HSPs), evolutionarily conserved molecular chaperones, are critical for alleviating cellular stress, tempering inflammatory responses, and restoring tissue homeostasis [11]. In the context of renal pathophysiology, HSPs, such as heat shock protein 70 (HSP70) are critical for attenuating ischemia-reperfusion (I/R) injury by preserving renal cell viability and function [12]. Beyond its canonical role in protein quality control, HSP70 is a pivotal mediator of inflammation, with demonstrated efficacy in reducing apoptosis and renal damage in S-AKI models [13, 14]. Notably, HSP70-mediated signaling pathways—including STAT3 activation—have been implicated in macrophage polarization, driving a phenotypic shift from proinflammatory M1 (marked by CD68) to anti-inflammatory M2 (marked by CD163) states in conditions, such as drug-induced AKI [15, 16]. This functional reprograming of macrophages underscores HSP70's dual role as both a cytoprotective agent and an immunomodulator. The TPR chaperone heat shock binding protein 21(HBP21)/TTC36 regulates HSP70 via direct binding, fine-tuning its molecular activity [17]. While earlier studies established HBP21 as a tumor suppressor in hepatocellular and gastric carcinomas [18, 19], recent work revealed its involvement in innate immunity, where it enhances interferon regulatory factor 3 (IRF3) activation during antiviral responses [20]. Despite these advances, the potential interplay between HBP21 and macrophage polarization remains unexplored. Given HSP70's established role in modulating macrophage phenotypes, HBP21—as its chaperone—may serve as a critical upstream regulator of this process, particularly in inflammatory pathologies like S-AKI. However, the mechanisms by which HBP21 influences macrophage activation or resolves septic renal injury have yet to be elucidated.

Therefore, this work focuses on examining the impacts of HBP21 in S-AKI pathogenesis using LPS-induced macrophage and cecal ligation and puncture (CLP)-induced rat AKI models, with a focus on its regulation of macrophage polarization and the PI3K/AKT signaling pathway.

## 2. Materials and Methods

### 2.1. Cell Culture and Treatments

RAW264.7 macrophages (ATCC TIB-71) and NRK-52E epithelial cells (ATCC CRL-1571) were grown in DMEM (D6046, Sigma-Aldrich) with 10% FBS and 1% penicillin–streptomycin (Gibco) at 37°C/5% CO_2_. RAW264.7 macrophages were polarized toward an M1 phenotype using *E. coli* 011: B4 LPS (Sigma-Aldrich) across a dose gradient (0–1000 ng/ml) and time course (0–24 h). These concentrations were selected based on literature consensus and time-dependent activation kinetics (24 h reflects peak M1 marker expression). RAW264.7 cells were pretreated with PI3K modulators (LY294002 inhibitor/740Y-P agonist, 10 μM) for 1 h before LPS challenge (1000 ng/ml, 24 h) to dissect HBP21's regulation of PI3K/AKT-dependent macrophage polarization.

### 2.2. Cell Transfection

Ov-HBP21 (overexpressing adenovirus), si-HBP21 (knockdown), and controls (Ov-NC/si-NC) were sourced from Gene Pharma. Transfection into RAW264.7 cells (1 μg, 24 h) utilized Lipofectamine 2000 (Invitrogen) per manufacturer guidelines.

### 2.3. Flow Cytometric Analysis

M1/M2 surface markers were assessed by flow cytometry. Harvested cells (5 × 10^5^) underwent trypsinization, centrifugation (1000 rpm, 5 min), and PBS washes. Pellets were stained with anti-CD86 (ab239075) and anti-CD206 (ab270634; 1:500, Abcam) for 20 min (4°C/dark), followed by FITC-conjugated secondary antibodies (ab150077) for 40 min. Cells were resuspended in PBS and analyzed using a FACSCalibur (BD Biosciences).

### 2.4. Coculture Model

A transwell coculture system was used to study interactions between NRK-52E cells and RAW264.7 macrophages. In brief, 1 ml of NRK-52E cells (1 × 10^4^ cells), either induced by LPS or without LPS induction, were seeded at the bottom of a 24-well plate. After 4 h, 1 ml of RAW264.7 macrophages (1 × 10^4^ cells) from LPS + Ov-NC, LPS + Ov-HBP21, and LPS + Ov-HBP21 + LY294002 groups were added to the upper chamber. After 24 h of coincubation, NRK-52E cells were harvested for further analysis. Three biological replicates were conducted.

### 2.5. Cell Viability Assay

NRK-52E cell viability was assessed using a CCK-8 assay kit (Beyotime). Cells (4.0 × 10^3^) were plated in 96-well plates with DMEM and incubated for 24–72 h. CCK-8 reagent (5 µl) was added for 2 h at 37°C, followed by absorbance measurement at 450 nm on a BioTek microplate reader. Three biological replicates were conducted.

### 2.6. Apoptosis Assay

Apoptosis in NRK-52E cells was quantified via Annexin V/PI staining (KeyGEN KGA1101-20). Following trypsinization and centrifugation (1000 rpm, 5 min, 4°C), cells (1 × 10^6^/ml) were stained with Annexin V-FITC/PI (5 µl each, 15 min, RT/dark) and analyzed by flow cytometry (BD FACSCalibur; triplicate experiments).

### 2.7. Caspase-3 Activity Assay

Caspase-3 activity, a marker of apoptosis, was quantified using a colorimetric assay kit (Abcam) following the manufacturer's protocol. Absorbance at 400 nm was measured with a BioTek microplate reader, and values were normalized to the control group. Three biological replicates were conducted.

### 2.8. Animal Experiments

Male Sprague–Dawley rats (8–9 weeks, 180–210 g) sourced from Ziyuan Laboratory Animal Technology (China) were maintained in SPF conditions (23 ± 2°C) with free access to food and water. Rats were randomly divided into four groups (*n* = 6 each): sham, S-AKI model, S-AKI + Ov-NC, and S-AKI + Ov-HBP21. S-AKI rat models were generated via CLP, following established protocols [21]. In the sham group, the abdomen of the rats was promptly closed after laparotomy without performing cecal ligation or puncture. Ov-HBP21 or Ov-NC (1 × 10^9^/ml, 100 µl) was delivered via tail vein to assess HBP21's impact on S-AKI in rats [22]. Postoperative care included hydration (5 ml/100 g saline, 37°C) and analgesia (0.05 mg/kg buprenorphine). After 24 h, rats were anesthetized (3% pentobarbital) and euthanized. Serum was isolated from centrifuged blood (10,000 rpm, 10 min, 4°C), and kidneys were collected for analysis. All animal procedures adhered to ARRIVE guidelines and were approved by the Ethics Committee of Hainan Affiliated Hospital of Hainan Medical University (Approval No. 2023103, Hainan, China).

### 2.9. Assessment of Renal Function

Scr (C011-2-1) and blood urea nitrogen (BUN, C013-2-1) levels were measured using manufacturer-specified kits (Nanjing Jiancheng) and a Vitros V5600 analyzer (Johnson).

### 2.10. Enzyme-Linked Immunosorbent Assay (ELISA)

TNF-α, IL-1β, and IL-6 were measured in mouse (RAW264.7) and rat (serum) samples using BOSTER ELISA kits (mouse: EK0527, EK0394, and EK0411; rat: EK0526, EK0393, and EK0410) per manufacturer guidelines.

### 2.11. Histopathological Analysis

The removed kidney tissues were initially rinsed with sterile saline. Subsequently, they were fixed using 4% paraformaldehyde and dehydrated through an ethanol gradient comprising sequential steps of 70%, 85%, 95%, and 100% ethanol. Thereafter, tissue samples were processed into 4-μm paraffin sections. Kidney sections were dewaxed in xylene, stained with hematoxylin and eosin (H&E) using a commercial kit (Beyotime, C0105M), and examined under an optical microscope (× 200 magnification). Tubular damage was assessed via a double-blind, randomized approach using a predefined scoring system: 0 (<10%), 1 (10%–25%), 2 (25%–50%), 3 (50%–75%), and 4 (>75%). Average scores per visual field were statistically analyzed to quantify severity.

### 2.12. Terminal dUTP Nick-End Labeling (TUNEL) Assay

Apoptotic cells in renal tissues were identified using a TUNEL Apoptosis Assay kit (Beyotime). Briefly, sections underwent deparaffinization through three 5-min xylene immersions. Subsequently, sections were treated with 20 μg/ml protease K (15 min, room temperature), incubated with 50 μl TUNEL solution (1 h, 37°C in the dark), washed with PBS, and sealed using anti-fluorescence quenching solution. Nuclei were stained blue with DAPI, and images (×200 magnification) were captured using an Olympus fluorescence microscope. TUNEL-positive cells were quantified with ImageJ software.

### 2.13. Immunohistochemistry

The expression of HBP21 was determined through an immunohistochemistry assay. First, kidney sections underwent deparaffinization, rehydration, and endogenous peroxidase inactivation (3% H_2_O_2_). Subsequently, the sections were preincubated for 1 h with 1.5% BSA and a primary antibody against HBP21 (1:500, ab122507, Abcam). After secondary antibody treatment (1% BSA), 3,3′-diaminobenzidine (DAB, Beyotime) staining and hematoxylin counterstaining (2 min) were performed, followed by fluorescence microscopy (Olympus).

### 2.14. Western Blot Analysis

Protein was isolated from cells and kidney tissues with RIPA buffer (Beyotime), and concentrations were measured via BCA assay. Protein samples (30 µg/lane) were separated by 12% SDS-PAGE, transferred to PVDF membranes, and blocked with 5% skim milk in TBST (2 h, room temperature). Membranes were incubated overnight at 4°C with primary antibodies: HBP21 (1:1000, ab122507; Abcam), iNOS (#13120), Arg-1 (#93668), Bcl-2 (#15071), cleaved caspase-3 (#9664), p-PI3K (#4228), PI3K (#4292), p-AKT (1:2000, #4060), AKT (#9272), and GAPDH (#2118) (all 1:1000 unless specified; Cell Signaling Technology), with GAPDH serving as the loading control. Post-wash, membranes were incubated with HRP-conjugated secondary antibodies (1:5000, #7074, cell signaling) for 1.5 h at room temperature. Protein bands were visualized using enhanced chemiluminescence (Solarbio) and analyzed via ImageJ densitometry.

### 2.15. Statistical Analysis

Results were expressed as means ± SD from ≥3 independent experiments. Statistical analyses were performed in GraphPad Prism 8.0, with group comparisons conducted via unpaired Student's *t*-test or one-way ANOVA, followed by Tukey's or Bonferroni's post hoc tests where appropriate. A *p*-value <0.05 was considered statistically significant.

## 3. Results

### 3.1. HBP21 Expression Reduction During M1 Polarization in LPS-Stimulated RAW264.7 Cells

**Figure 1:**
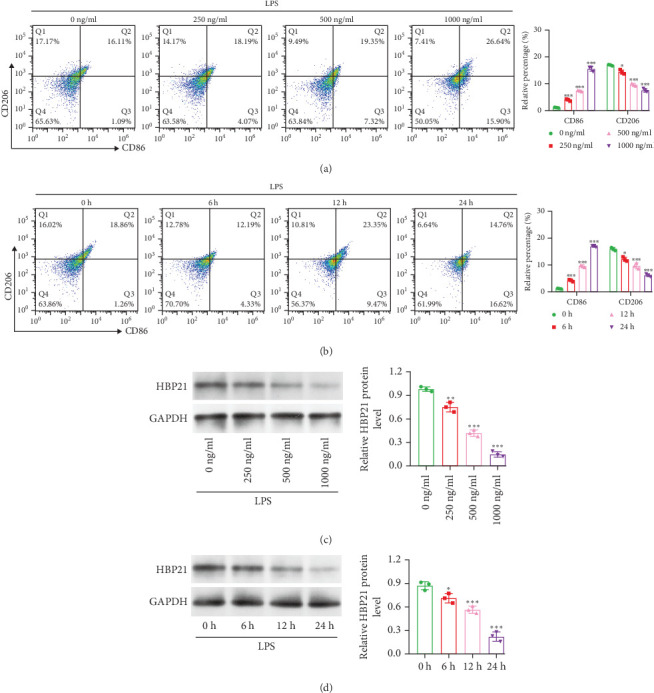
HBP21 expression reduction during M1 polarization in LPS-stimulated RAW 264.7 cells. Flow cytometry analysis of RAW 264.7 macrophages labeled with CD86 and CD206 exposed to different concentrations of LPS (0, 250, 500, and 1000 ng/ml) (A) and to 1000 ng/ml LPS for different times (0, 6, 12, and 24 h) (B). Western blot analysis showed the downregulation of HBP21 protein after LPS treatment in a dose-dependent (C) and time-dependent (D) manner. Data were presented as means ± SD. *⁣*^*∗*^*p* < 0.05, *⁣*^*∗∗*^*p* < 0.01, *⁣*^*∗∗∗*^*p* < 0.001, compared with 0 ng/ml or 0 h, via one-way ANOVA analysis, followed by Bonferroni's post hoc test.

Initially, RAW264.7 macrophages were polarized toward an M1 phenotype using LPS in dose (0–1000 ng/ml, 24 h) and time-dependent (0–24 h, 1000 ng/ml) experiments. Flow cytometry assessed CD86 (M1) and CD206 (M2) expression. LPS dose-dependently increased M1 macrophages (CD86^+^) while decreasing M2 macrophages (CD206^+^) in RAW264.7 cells (Figure 1A). Time-dependent enhancement of M1 polarization and reduction of M2 polarization were further confirmed at 1000 ng/mL LPS (Figure 1B). Western blot analysis revealed LPS-mediated downregulation of HBP21 expression in dose- (Figure 1C) and time-dependent (Figure 1D) manners. Based on these findings, 1000 ng/ml LPS and a 24-h treatment duration were selected for further experiments.

### 3.2. Overexpression of HBP21 Attenuated the Inflammation and M1 Polarization in LPS-Stimulated RAW 264.7 Cells

**Figure 2:**
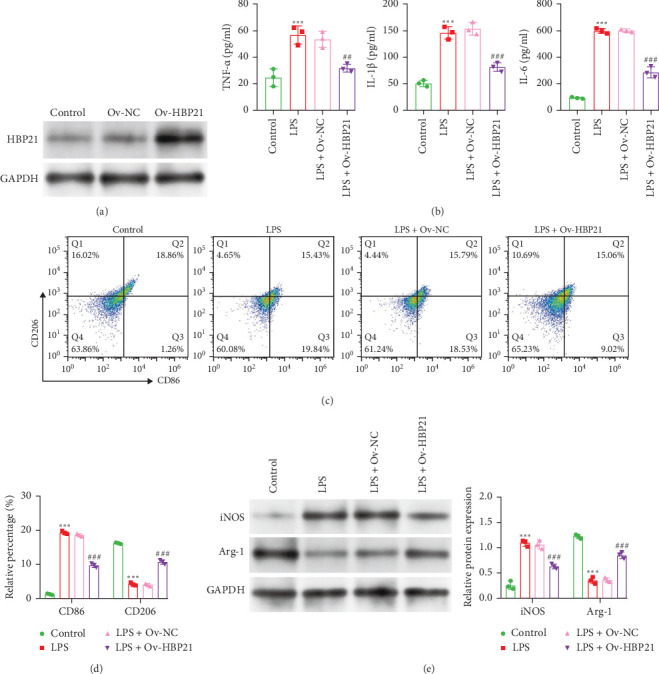
Overexpression of HBP21 attenuated the inflammation and M1 polarization in LPS-stimulated RAW 264.7 cells. (A) Western blot analysis was utilized to determine the transfection efficiency of Ov-HBP21 in LPS-induced RAW 264.7 macrophages. (B) Levels of TNF-α, IL-1β, and IL-6 in LPS-induced RAW 264.7 macrophages were evaluated using ELISA assay kits. (C and D) Flow cytometry analysis was performed on RAW 264.7 macrophages labeled with CD86 and CD206 in LPS-induced RAW 264.7 macrophages. (E) Representative images and quantification of iNOS and Arg-1 expression were obtained using western blot assay. Data were presented as means ± SD. *⁣*^*∗∗∗*^*p* < 0.001, compared with control via unpaired Student's *t*-test; *⁣*^##^*p* < 0.01, *⁣*^###^*p* < 0.001, compared with LPS + Ov-NC via unpaired Student's *t*-test.

To investigate HBP21's role in LPS-stimulated macrophage polarization, overexpression was achieved via Ov-HBP21 adenovirus transfection. In LPS-stimulated RAW264.7 macrophages, HBP21 expression was markedly upregulated in the Ov-HBP21 group compared to Ov-NC controls (Figure 2A). ELISA revealed that HBP21 overexpression significantly reduced TNF-α, IL-1β, and IL-6 levels in LPS-stimulated RAW264.7 macrophages (Figure 2B). Flow cytometry analysis demonstrated that HBP21 overexpression most potently inhibited LPS-induced M1 polarization in RAW 264.7 cells (Figure 2C,D). Ov-HBP21 also suppressed LPS-induced M1 polarization by reducing iNOS expression while upregulating the M2 marker Arg-1 (Figure 2E). Together, these results indicate HBP21 promotes a shift from LPS-triggered M1 to M2 polarization in RAW 264.7 macrophages.

### 3.3. HBP21 Exerted Suppressive Impacts on LPS-Induced Macrophage M1 Polarization via PI3K/AKT Pathway Activation

**Figure 3:**
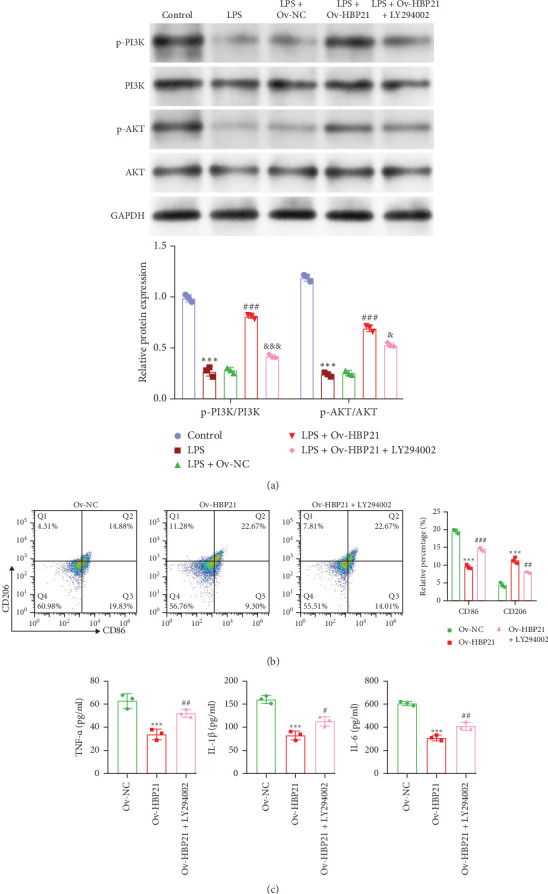
HBP21 exerted suppressive impacts on LPS-induced macrophage M1 polarization via PI3K/AKT pathway activation. RAW 264.7 cells were transfected with Ov-HBP21 for 24 h, then pretreated with the PI3K inhibitor LY294002 for 1 h prior to the addition of LPS for 24 h. (A) The protein expression of p-PI3K, PI3K, p-AKT, and AKT was detected in the above RAW 264.7 cells. *⁣*^*∗∗∗*^*p* < 0.001, compared with control; ###*p* < 0.001, compared with LPS + Ov-NC; *⁣*^&^*p* < 0.05, *⁣*^&&^*p* < 0.01, *⁣*^&&&^*p* < 0.001, compared with LPS + Ov-HBP21 via one-way ANOVA analysis, followed by Tukey's post hoc test; (B) Flow cytometry analysis was performed on the above RAW 264.7 cells labeled with CD86 and CD206 in the above RAW 264.7 cells. (C) Levels of TNF-α, IL-1β, and IL-6 in the above RAW 264.7 cells were evaluated using ELISA assay kits. *⁣*^*∗∗∗*^*p* < 0.001, compared with Ov-NC; *⁣*^#^*p* < 0.05, *⁣*^##^*p* < 0.01, *⁣*^###^*p* < 0.001, compared with Ov-HBP21 via one-way ANOVA analysis, followed by Tukey's post hoc test. Data were presented as means ± SD.

The PI3K/AKT pathway contributes to sepsis-associated renal injury. To evaluate the regulatory role of HBP21 in this pathway, RAW 264.7 macrophages transfected with Ov-HBP21 were pretreated with the PI3K inhibitor LY294002 prior to LPS stimulation. LY294002 attenuated LPS-induced phosphorylation of PI3K and AKT in Ov-HBP21-transfected cells, as demonstrated by western blot (Figure 3A). Flow cytometry further demonstrated that inhibiting the PI3K/AKT pathway reversed HBP21-mediated polarization shifts, marked by elevated CD86 (M1) and reduced CD206 (M2) levels compared to the LPS + Ov-HBP21 group (Figure 3B). Consistently, LY294002 significantly increased TNF-α, IL-1β, and IL-6 levels in LPS-treated HBP21-overexpressing macrophages (Figure 3C). Complementary experiments using HBP21 knockdown (si-HBP21) and the PI3K agonist 740 Y-P were conducted to validate these findings. Transfection efficiency confirmed significant HBP21 downregulation in si-HBP21-transfected macrophages (Figure S1A). Notably, 740 Y-P restored p-PI3K and p-AKT levels suppressed by HBP21 silencing (Figure S1B). Flow cytometry showed that PI3K/AKT activation via 740 Y-P counteracted the effects of HBP21 knockdown, reducing CD86 and elevating CD206 expression (Figure S1C). Correspondingly, 740 Y-P lowered TNF-α, IL-1β, and IL-6 levels in LPS-treated si-HBP21 macrophages (Figure S1D). These findings suggest HBP21 facilitates M2 polarization and mitigates LPS-driven inflammation via PI3K/AKT pathway activation.

### 3.4. Overexpression of HBP21 Alleviated LPS-Triggered Tubular Epithelial Cell Damage Through PI3K/AKT Activation

**Figure 4:**
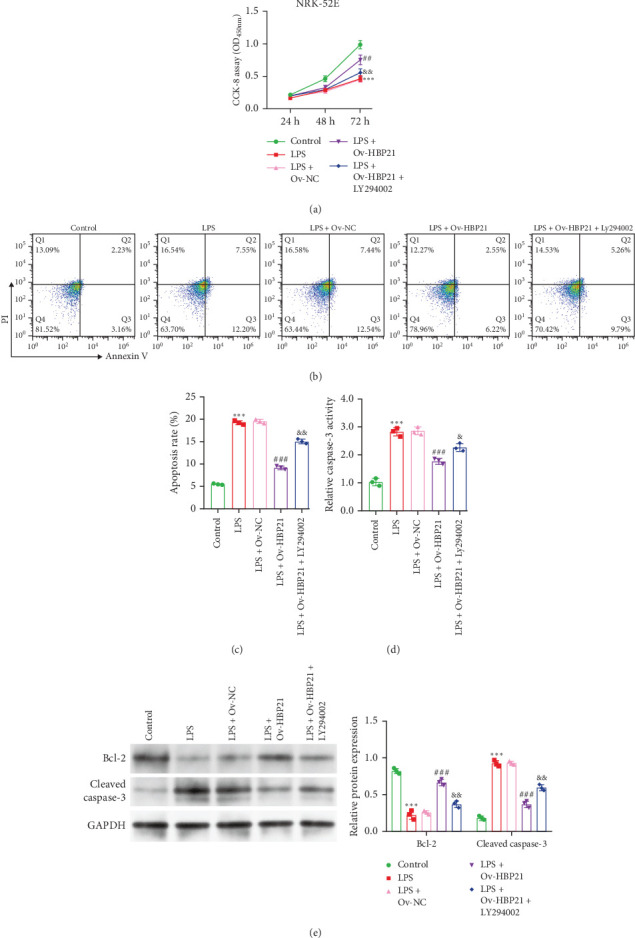
Overexpression of HBP21 alleviated LPS-triggered tubular epithelial cell damage through PI3K/AKT activation. NRK-52E cells were cocultured with RAW264.7 macrophages from the control, LPS, LPS + Ov-NC, LPS + Ov-HBP21, and LPS + Ov-HBP21 + LY294002 groups for 24 h. (A) CCK-8 assay was utilized to analyze cell viability of NRK-52E cells from coculture system. (B and C) Cell apoptosis was determined by flow cytometry assay in NRK-52E cells from coculture system. (D) Relative caspase-3 activity was measured in NRK-52E cells from coculture system. (E) The protein expression of Bcl-2 and cleaved caspase-3 was detected in NRK-52E cells from coculture system. Data were presented as means ± SD. *⁣*^*∗∗∗*^*p* < 0.001, compared with control; *⁣*^##^*p* < 0.01, *⁣*^###^*p* < 0.001, compared with LPS + Ov-NC; *⁣*^&^*p* < 0.05, *⁣*^&&^*p* < 0.01, compared with LPS + Ov-HBP21 via one-way ANOVA analysis, followed by Tukey's post hoc test.

To elucidate the role of HBP21-mediated PI3K/AKT signaling activation in LPS-induced renal tubular epithelial cell injury, coculture experiments were conducted using NRK-52E cells and RAW264.7 macrophages. Macrophages were allocated into five experimental groups: control, LPS-treated, LPS + Ov-NC, LPS + Ov-HBP21, and LPS + Ov-HBP21 + LY294002. Subsequent analyses evaluated the interplay between HBP21 overexpression and PI3K/AKT pathway modulation on the viability and apoptosis of NRK-52E cells. LPS stimulation markedly decreased cell viability compared to controls in NRK-52E cells (Figure 4A). Conversely, HBP21 overexpression in macrophages attenuated this LPS-induced cytotoxicity, restoring NRK-52E viability. Notably, this protective effect was partially reversed by LY294002 pretreatment, as evidenced by reduced viability in the LPS + Ov-HBP21 + LY294002 group relative to the LPS + Ov-HBP21 group. Apoptosis assays revealed that LPS exposure markedly increased the apoptotic rate of NRK-52E cells (Figure 4B,C). HBP21 overexpression suppressed this apoptotic response, whereas LY294002 cotreatment abolished the antiapoptotic effects of HBP21, restoring apoptosis to levels comparable to the LPS group. Consistent with these findings, caspase-3 activity assays demonstrated elevated enzymatic activity in LPS-treated cells, which was mitigated by HBP21 overexpression and reinstated by LY294002 (Figure 4D). Western blot analysis further corroborated these results (Figure 4E). HBP21 overexpression promoted Bcl-2 (antiapoptotic) while inhibiting cleaved caspase-3 in LPS-stimulated NRK-52E cells. However, LY294002 pretreatment neutralized these regulatory effects, implicating PI3K/AKT signaling as a critical mediator of HBP21's protective role.

### 3.5. Overexpression of HBP21 Improved the Renal Function and Ameliorated Pathological Injury in S-AKI Rat

**Figure 5:**
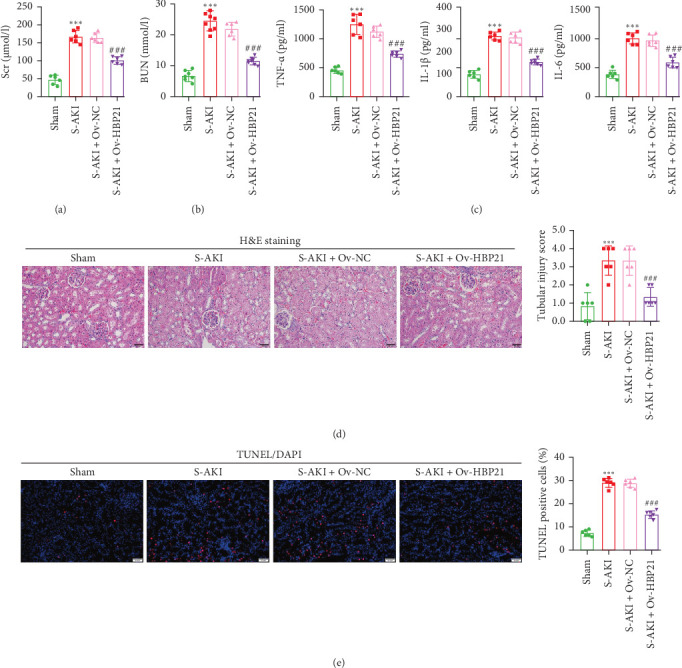
Overexpression of HBP21 improved the renal function and ameliorated pathological injury in S-AKI rats. A rat model of S-AKI was developed, and either Ov-HBP21 or Ov-NC was administered to this model (*n* = 6 each group). (A) Levels of Scr and (B) Levels of BUN. (C) Serum levels of inflammatory factors TNF-α, IL-1β, and IL-6. (D) Hematoxylin and eosin (H&E) staining images of rat kidney tissues from each group. Tissue damages were scored based on pathological results and analyzed through a histogram. Scale bar: 50 μm. (E) TUNEL staining of renal tissues and quantification of TUNEL-positive cells in each group. Bars = 50 μm. Data were presented as means ± SD. *⁣*^*∗∗∗*^*p* < 0.001, compared with sham; *⁣*^###^*p* < 0.001, compared with S-AKI + Ov-NC via unpaired Student's *t*-test.

To confirm the renal protective effects of HBP21 in vivo, a rat S-AKI model was generated, followed by intrarenal delivery of Ov-HBP21 or Ov-NC. Serum analysis showed elevated kidney injury markers (Scr, BUN; Figure 5A,B) and proinflammatory cytokines (TNF-α, IL-1β, IL-6; Figure 5C) in S-AKI rats compared to sham controls. However, HBP21 overexpression via Ov-HBP21 administration markedly attenuated these pathological changes, reducing Scr, BUN, and cytokine levels to near-baseline values. Histopathological examination further demonstrated the therapeutic potential of HBP21. Renal tissues from S-AKI rats displayed severe structural damage, characterized by brush border loss, vacuolar degeneration, and tubular cast formation (Figure 5D). In contrast, HBP21-overexpressing S-AKI rats exhibited significant amelioration of these histological abnormalities, with preserved tubular architecture. Apoptotic responses were quantified using TUNEL staining (Figure 5E). S-AKI rats exhibited markedly elevated renal cell apoptosis versus sham controls, whereas Ov-HBP21 treatment substantially suppressed apoptotic activity. Together, these findings indicate that HBP21 overexpression mitigated renal dysfunction, inflammation, structural damage, and apoptosis in S-AKI, underscoring its protective role in septic kidney injury.

### 3.6. Overexpression of HBP21 Attenuated Kidney Macrophage M1 Polarization and Activated PI3K/AKT Signaling in S-AKI Rats

**Figure 6:**
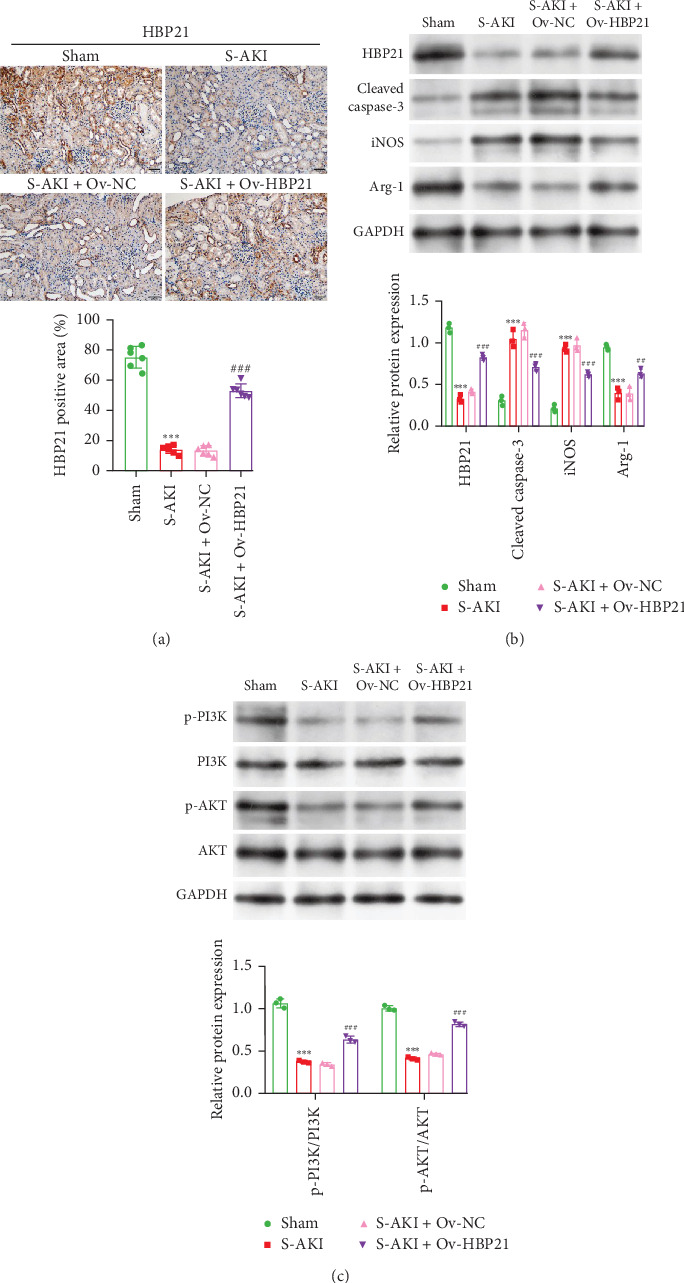
Overexpression of HBP21 attenuated kidney macrophage M1 polarization and activated PI3K/AKT signaling in S-AKI rats. An S-AKI rat model was established, and either Ov-HBP21 or Ov-NC was administered to this model (*n* = 6 per group). (A) Photomicrographs of kidney tissue sections immunohistochemically stained for HBP21, along with quantification of the HBP21-positive staining area. (B) Western blot analysis was employed to detect HBP21, cleaved caspase-3, iNOS, and Arg-1 in the kidney tissues. (C) Western blot analysis was used to detect p-PI3K, PI3K, p-AKT, and AKT in the kidney tissues. Data were presented as means ± SD. *⁣*^*∗∗∗*^*p* < 0.001, compared with sham; *⁣*^##^*p* < 0.01, *⁣*^###^*p* < 0.001, compared with S-AKI + Ov-NC via unpaired Student's *t*-test.

To further characterize the functional role of HBP21 in S-AKI, we analyzed its expression and functional impact in renal tissues from S-AKI rats. Immunohistochemistry revealed significantly reduced HBP21 expression in S-AKI rat kidneys versus sham controls. This reduction was rescued by Ov-HBP21 administration, confirming successful HBP21 overexpression in the treatment group (Figure 6A). We next assessed the influence of HBP21 on apoptosis, macrophage polarization, and PI3K/AKT signaling. Renal tissues from S-AKI rats displayed increased cleaved caspase-3 and iNOS levels with reduced HBP21 and Arg-1 expression. HBP21 overexpression reversed these changes, attenuating apoptosis and promoting anti-inflammatory M2 macrophage polarization (Figure 6B). Western blot analysis further demonstrated that S-AKI induced significant inhibition of the PI3K/AKT pathway, as evidenced by reduced phosphorylation of key pathway components. However, Ov-HBP21 treatment restored PI3K/AKT activation, suggesting HBP21-mediated rescue of this critical signaling axis (Figure 6C). These findings suggest that overexpression of HBP21 mitigated S-AKI by diminishing renal macrophage M1 polarization and reactivating the PI3K/AKT signaling.

## 4. Discussion

S-AKI is a prevalent and grave complication in sepsis, resulting in an elevated risk of mortality [23]. Although significant progress has been made over the past decade, the genetic and molecular interdependence between inflammation and tubular damage during S-AKI remains poorly defined. Here, we first noted HBP21 downregulation during LPS-induced M1 polarization in RAW264.7 cells (a widely used murine monocyte/macrophage model), with dose- and time-dependent effects. HBP21 overexpression suppressed LPS-induced macrophage M1 polarization and inflammation while enhancing M2 polarization. We further established a CLP-induced S-AKI rat model, which displayed elevated Scr, BUN, renal tubular injury, interstitial macrophage infiltration, and heightened proinflammatory cytokines (IL-6 and TNF-α) [24]. Consistently, overexpression of HBP21 effectively alleviated the pathological injury in S-AKI rats and attenuated kidney macrophage M1 polarization. Our data indicate HBP21 is critical in S-AKI development and progression.

HBP21, an HSP70 chaperone, interacts with this protein to block PP2A-IRF3 association, thereby enhancing antiviral innate immunity [20]. HSP70 is regarded as a significant protein because of its advantageous effects in counteracting oxygen-free radicals, inflammation, and stress response [25, 26]. In accordance with our data, HSP70 ameliorates S-AKI by attenuating renal dysfunction, tissue damage, and inflammatory cascades while improving survival. This protective effect is mediated through suppression of NF-κB signaling and inflammation-driven apoptosis [14]. HSP70 activation attenuates ischemic acute renal failure via blockade of NF-κB inflammation and cell death cascades [27]. Extracellular HSP70 alleviates myocardial and hepatic dysfunction by suppressing inflammatory mediators (e.g., TNF-α and iNOS) through the MAPK/NF-κB pathway [28]. HSP70 inhibits p65 nuclear translocation in LPS-stimulated macrophages and dendritic cells, suppressing inflammatory responses [29]. Considering the correlation between HBP21 and HSP70, we propose HBP21 mitigates S-AKI by reducing renal injury and inflammation via inhibition of M1 macrophage polarization.

Several investigations have demonstrated that macrophage-driven inflammation and renal tubular epithelial cell death critically interact to regulate AKI pathogenesis and recovery [30, 31]. Our current data showed that HBP21 attenuated rat renal tubular epithelial cell apoptosis by shifting macrophage polarization from M1 to M2. This effect, confirmed in vivo, occurred following HBP21 overexpression in CLP-induced rats. As far as we know, renal tubular cells are major constituents of kidney parenchyma and are susceptible to multiple insults. In S-AKI, renal tubular epithelial cells are damaged and their death, including apoptosis, ferroptosis, and necroptosis, is the primary cause of the rapid progression of AKI diseases [32, 33]. Meanwhile, activated macrophages upregulate the M1 marker iNOS in S-AKI [34]. Similar to our research, Shao et al. [35] recently observed that dehydroandrographolide mitigates renal tubular epithelial cell apoptosis and inflammatory damage by shifting M1 to M2 polarization in THP-1-derived macrophages. This finding supports our results and implies that overexpression of HBP21 in RAW264.7 macrophages alleviates LPS-induced kidney tubular epithelial cell injury.

The PI3K/AKT pathway regulates inflammatory responses and cellular homeostasis, critically mitigating organ injury in sepsis and I/R [36–38]. In this study, we demonstrate that HBP21 overexpression activates PI3K/AKT signaling in LPS-stimulated RAW264.7 macrophages, driving a phenotypic shift from proinflammatory M1 (CD86^+^) to anti-inflammatory M2 (CD206^+^) polarization. This effect was reversed by the PI3K inhibitor LY294002, which also exacerbated LPS-induced tubular epithelial injury, underscoring the pathway's centrality in HBP21-driven immunomodulation and renal protection. These findings align with prior studies showing that PI3K/AKT activation reduces inflammation and organ damage in sepsis models. For instance, Qu et al. [39] reported the synthetic peptide HBSP (Helix B-Surface peptide) reduces LPS-induced renal injury via PI3K/AKT signaling. Similarly, Astragaloside IV (AS-IV) and the angiotensin receptor agonist C21, both exogenous compounds, exert renoprotective effects through the same pathway in septic and CLP models, respectively [40, 41]. However, unlike these pharmacologic agents, HBP21 represents an endogenous regulator of PI3K/AKT signaling, suggesting its unique potential as a physiologically compatible therapeutic target in S-AKI. Notably, HSP70—a molecular chaperone directly interacting with HBP21-has been implicated in macrophage polarization through STAT3-dependent mechanisms, favoring M2 transitions in drug-induced AKI [15, 16]. While our study did not directly investigate HSP70, the parallels between its known immunomodulatory functions and HBP21's effects on macrophage reprograming suggest a coordinated regulatory axis. As a chaperone, HBP21 may stabilize HSP70's conformation, thereby enhancing its capacity to suppress proinflammatory cytokines and promote tissue repair [18]. This hypothesis is supported by evidence that HSP70 requires chaperones like HBP21 to optimize its function in stress responses [42]. Thus, HBP21's activation of PI3K/AKT signaling could complement HSP70-driven STAT3 activation, creating a synergistic mechanism to resolve inflammation in S-AKI.

While our study identifies HBP21 as a potential therapeutic target for S-AKI, several limitations must be acknowledged. First, the study relied on murine macrophages (RAW264.7) and rat renal epithelial cells, which may not fully recapitulate human immune responses or sepsis pathophysiology. Second, our focus on the PI3K/AKT pathway does not exclude contributions from other signaling cascades (e.g., NF-κB, JAK/STAT) that may interact with HBP21 in modulating macrophage polarization. Third, the acute LPS-induced AKI model does not mirror the heterogeneity of human sepsis, where comorbidities, genetic variability, and polymicrobial infections complicate therapeutic outcomes. Finally, the translational potential of HBP21 is constrained by its physicochemical properties (e.g., stability and bioavailability) and the lack of data on its safety in primates or human cells. Despite these limitations, the clinical implications of our work are significant. The PI3K/AKT pathway is a conserved therapeutic target across species, and HBP21's endogenous nature may reduce immunogenicity risks compared to synthetic agents like HBSP or AS-IV [40, 41]. To bridge the translational gap, future clinical studies should: (1) conduct Phase I trials to evaluate HBP21's safety, pharmacokinetics, and dosing in humans; (2) validate PI3K/AKT-mediated macrophage polarization in sepsis patient cohorts using single-cell RNA sequencing or flow cytometry of blood/tissue samples; (3) design biomarker-driven Phase II trials to assess HBP21's efficacy in reducing AKI incidence and cytokine storms (e.g., TNF-α and IL-6) in septic patients; (4) explore HBP21 in combination with standard therapies (e.g., antibiotics and vasopressors) to enhance survival rates; and (5) establish long-term renal safety and functional recovery metrics in late phase trials. Companion diagnostics targeting PI3K/AKT dysregulation (e.g., phospho-AKT assays and macrophage polarization signatures) could further enable precision patient stratification.

## 5. Conclusion

Our findings demonstrate that HBP21 alleviates S-AKI in vitro and in vivo, likely by promoting M2 macrophage polarization via PI3K/AKT pathway activation. This work advances understanding of S-AKI pathogenesis and highlights HBP21 as a potential therapeutic target.

## Data Availability

All data from this study are available from the corresponding author upon request.

## Supporting Information

Supporting Information Figure S1: PI3K/AKT pathway activation rescued HBP21 knockdown effects in LPS-stimulated RAW264.7 macrophages. (A) Western blot analysis validation of HBP21 protein levels in si-HBP21-transfected cells. (B) Western blot analysis of p-PI3K and p-AKT expression in si-HBP21 cells pretreated with 740 Y-P for 1 h prior to the addition of LPS for 24 h. (C) Flow cytometry analysis of CD86 and CD206 expression. (D) ELISA quantification of TNF-α, IL-1β, and IL-6 levels. Data were presented as means ± SD. *⁣*^*∗∗*^*p* < 0.01, *⁣*^*∗∗∗*^*p* < 0.001, compared with LPS + si-NC; *⁣*^#^*p* < 0.05, *⁣*^##^*p* < 0.01, *⁣*^###^*p* < 0.001, compared with LPS + si-HBP21 via one-way ANOVA analysis, followed by Tukey's post hoc test.
