# Supplementary figures and images for: HBP21 Alleviates Sepsis-Induced Acute Kidney Injury by Targeting PI3K/AKT-Mediated M1 Macrophage Polarization
Source: Mediators Inflamm. 2025 Jul 27;2025:9021628. doi: 10.1155/mi/9021628 (PMC12318628; doi:10.1155/mi/9021628)

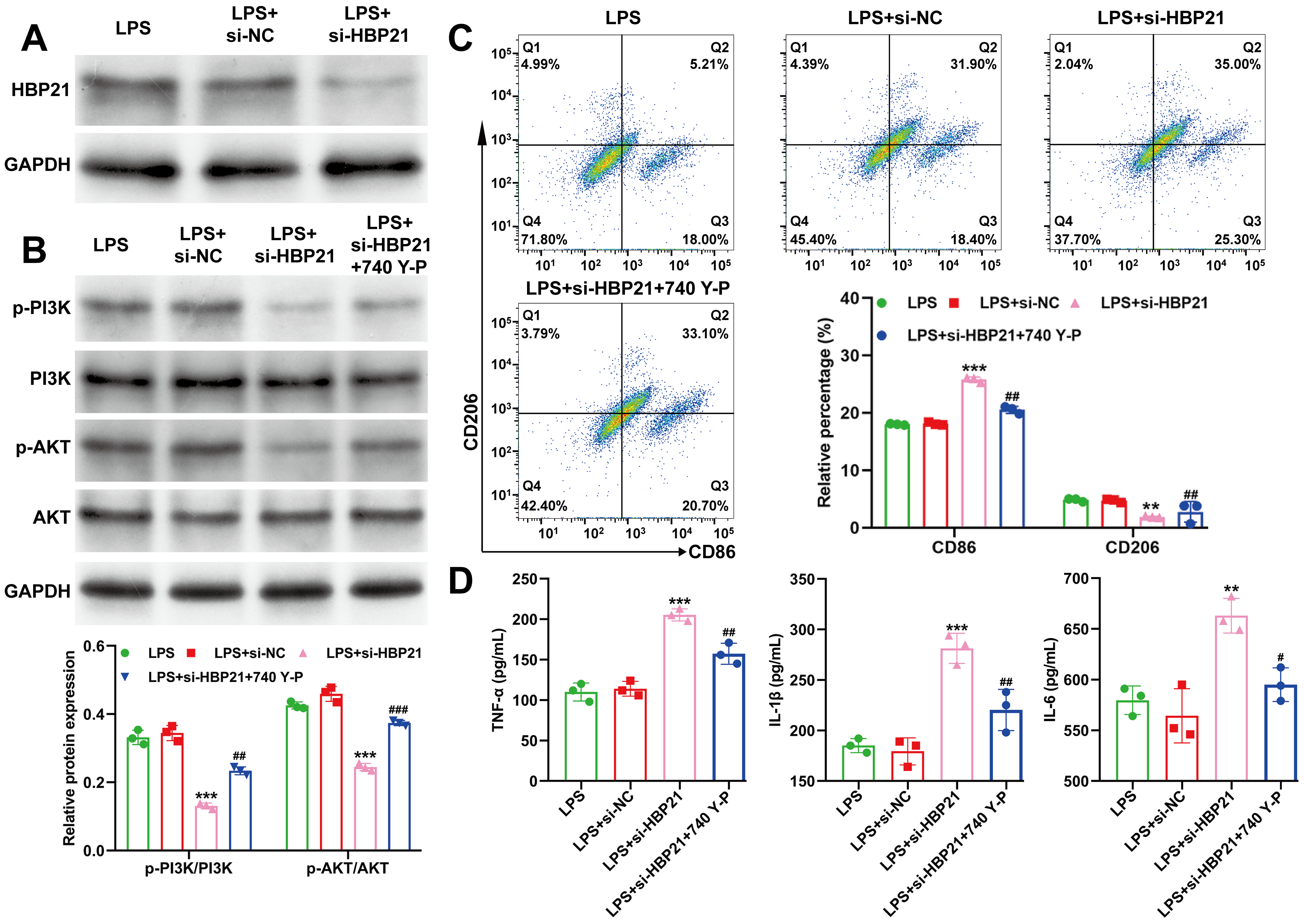

Supplement: Supporting Information — Figure S1: PI3K/AKT pathway activation rescued HBP21 knockdown effects in LPS-stimulated RAW264.7 macrophages. (A) Western blot analysis validation of HBP21 protein levels in si-HBP21-transfected cells. (B) Western blot analysis of p-PI3K and p-AKT expression in si-HBP21 cells pretreated with 740 Y-P for 1 h prior to the addition of LPS for 24 h. (C) Flow cytometry analysis of CD86 and CD206 expression. (D) ELISA quantification of TNF-α, IL-1β, and IL-6 levels. Data were presented as means ± SD. ⁣∗∗p < 0.01, ⁣∗∗∗p < 0.001, compared with LPS + si-NC; ⁣#p < 0.05, ⁣##p < 0.01, ⁣###p < 0.001, compared with LPS + si-HBP21 via one-way ANOVA analysis, followed by Tukey's post hoc test. [file 9021628.f1.tif]
